# Transcriptomics Analysis Reveals an Early Response Gene *SlNSP-like* Involved in *Solanum lycopersicum* Response to DC3000 Infection

**DOI:** 10.3390/cimb48010011

**Published:** 2025-12-22

**Authors:** Junqing Li, Mengjie Gu, Mengsen Yang, Huimin Tan, Wei Yang, Guanghui Qi

**Affiliations:** 1 College of Information Science and Engineering, Shandong Agricultural University, Tai’an 271018, China2025121193@sdau.edu.cn (M.Y.); 2 National Key Laboratory of Wheat Breeding, College of Life Science, Shandong Agricultural University, Tai’an 271018, China

**Keywords:** *Solanum lycopersicum*, *SlNSP-like*, *Pst* DC3000, transcriptome analysis, pathogen resistance

## Abstract

The hemibiotrophic bacterial pathogen *Pseudomonas syringae* (*Pst*) infects a range of plant species and causes enormous economic losses. Despite its agronomic significance, the molecular mechanisms underlying tomato–*Pst* interactions remain largely uncharacterized. To elucidate these mechanisms, we conducted a comprehensive transcriptomic analysis using infected tomato leaves inoculated with virulent strains *Pst* DC3000 at relatively early time points. RNA-sequencing of nine libraries identified stage-specific expression patterns, with DEG counts ranging from 484 to 1267 upregulated and from 560 to 844 downregulated genes. Enrichment analysis highlighted significant alterations in metabolic pathways, plant–pathogen interaction networks, and hormone signaling cascades, with marked transcriptional reprogramming observed between the pre- and post-infection stages. A longitudinal analysis of gene expression dynamics identified 15 consistently upregulated and 9 downregulated genes across all post-inoculation time points. Notably, in several candidate genes, a homologous gene of *AtNSP2*, *SlNSP-Like* was confirmed to be involved in disease resistance in tomato leaves. SlNSP-Like is localized in the cytoplasm and nucleus, and the transient overexpression of *SlNSP-Like* tomato plant exhibits significant resistance to *Pst* DC3000. This study provides valuable insights into the molecular dialogue between tomato and *Pst*, and the identified regulatory genes and pathways serve as promising targets for breeding disease-resistant tomato cultivars and developing management strategies against bacterial spot disease.

## 1. Introduction

Bacterial diseases, caused by pathogenic bacteria infecting plants, represent a major category of diseases in facility cultivation, with over 500 documented types. These diseases have become increasingly prevalent in recent years, affecting crops such as tomatoes, peppers, and cucumbers [1]. Among bacterial diseases, bacterial spot disease—caused by *Pseudomonas syringae* pv. tomato DC3000 (*Pst* DC3000)—is particularly devastating. This Gram-negative pathogen, a pathogenic variant of *Pseudomonas syringae*, primarily affects tomatoes and peppers in facility productions across regions [2]. First reported in the U.S. in 1993, it has since spread to over 20 countries, causing outbreaks and economic losses [3]. In northern China, it frequently reduces tomato yields by over 50% during severe outbreaks, hindering agricultural development [4]. Pst DC3000 overwinters in seeds, diseased residues, or soil and spreads via human activities, insects, or natural plant openings. As a biotrophic pathogen, it obtains nutrients directly from living cells, propagating intercellularly before invading tissues. Symptoms include dark brown/black spots with yellow halos on leaves, black stem lesions, and brown fruit spots [5,6].

As the most widely cultivated vegetable in controlled environments globally, tomatoes (*Solanum lycopersicum*) are prized for their nutrient-rich fruits, containing high levels of vitamins, lycopene, minerals, and unique flavors that make them a consumer favorite [7]. However, under facility cultivation conditions—characterized by high temperatures and humidity—tomato crops are highly susceptible to bacterial and fungal diseases. These pathogens thrive in such environments, causing severe outbreaks that lead to substantial economic losses in tomato production worldwide [8]. The interplay of warm, moist conditions accelerates pathogen proliferation, making disease management a critical challenge for sustainable facility agriculture [9].

*Pst* DC3000, a Gram-negative bacterial pathogen, holds significant scientific and agricultural value [10,11]. In 2003, it became the first *Pseudomonas syringae* strain to have its genome fully sequenced, revolutionizing the study of plant–pathogen interactions and pathogenic mechanisms [12]. As a model organism in plant molecular pathology, it serves as a cornerstone for investigating how pathogens invade and adapt to host plants [13]. Tomatoes, with their well-characterized genetics, ease of cultivation, and genetic tractability, are ideal model plants for such research [14]. The pathogen’s ability to cause widespread crop damage and economic losses has solidified its role as a key focus in agricultural and biological studies, driving innovations in disease control strategies and crop protection technologies to safeguard global food security.

RNA-sequencing (RNA-seq) is one of the common methods used to investigate the molecular mechanisms underlying pathogen resistance [15]. Based on this method, several key genes have been screened from multiple important crop species such as tomato, rice and maize [16,17,18,19]. In the study of tomato–DC3000 interaction, many functional genes have been reported, including, for example, some PTI and ETI pathway core response genes and hormone-signal-related response genes [5]. However, current omics research mostly focuses on a single dimension and lacks integrated analysis of genes, proteins, and metabolites. We aim to explore early responsive enzyme genes through transcriptomics and attempt to provide new insights into the interaction between DC3000 and tomato from a protein metabolic perspective. In this study, we investigated the global expression profile of tomato leaves during the early stages of *Pst* DC3000 attack. Based on the global expression analyses, we reveal several genes that respond to *Pst* infection. Importantly, the *SlNSP-Like* gene was characterized as a potential important gene governing tomato–*Pst* interactions, as confirmed through integrated plant physiological and molecular biological validation. The gene resources excavated in this study will contribute to the cultivation of resistant tomato varieties.

## 2. Materials and Methods

### 2.1. Plant Materials

Tomato (*Solanum lycopersicum* L., cv. Ailsa Craig (AC)) seeds were sown in trays containing a peat–vermiculite mixture (2:1, *v*/*v*) and incubated in growth chambers under controlled conditions: 23 °C/20 °C (day/night) with a 12-h photoperiod (8:00 a.m.–8:00 p.m.), photosynthetic photon flux density (PPFD) of 200 μmol m^−2^ s^−1^ from fluorescent lighting, and 70% relative humidity (RH). Seedlings were irrigated every three days and supplemented weekly with Hoagland nutrient solution to maintain optimal growth parameters.

### 2.2. Transcriptome Library Preparation and Sequencing

Three-week-old tomato leaves were harvested from plants at distinct temporal intervals post-inoculation with *Pst* DC3000, and then immediately cryopreserved in liquid nitrogen to maintain RNA integrity for subsequent extraction. Each experimental condition incorporated three independent biological replicates, with each replicate comprising a composite sample pooled from a minimum of three individual plants to enhance statistical robustness. All procedural steps—including poly(A) mRNA enrichment via oligo dT bead-based isolation, mRNA fragmentation into short sequences, adapter ligation, size-selective purification, PCR-mediated amplification, and high-throughput RNA sequencing (RNA-seq)—were executed by specialized technicians at Novogene Co., Ltd. (Beijing, China). Following poly(A) mRNA isolation, the transcripts were enzymatically cleaved into smaller fragments to facilitate library construction. A single-end RNA-seq library was generated for nine distinct treatment-derived samples and sequenced on the Illumina HiSeq™ 2000 platform, ensuring comprehensive transcriptional profiling across experimental conditions.

### 2.3. Quality Control of Raw Reads

The read quality of the raw transcriptome sequencing data was initially assessed using FastQC (v0.12.1; http://www.bioinformatics.babraham.ac.uk/projects/fastqc/ accessed on 7 August 2025). To remove adapter sequences and improve sequencing quality, raw reads were processed with fastp [20] (v1.0.1) for quality control and trimming. Key steps included automatic adapter identification and removal, quality-based trimming at read ends, removal of fragments shorter than 25 nt, and filtering of low-quality bases (retaining bases with a Phred quality score ≥ 20). The trimming process was accelerated using multi-threading.

Following trimming, FastQC was rerun on the processed reads to generate post-trimming quality reports. Finally, all sample-level FastQC reports were aggregated and visualized using MultiQC [21] (v1.28) to evaluate the effectiveness of the trimming procedure and assess the consistency of data quality across samples.

### 2.4. Read Alignment

The trimmed sequencing reads were aligned with the tomato reference genome (S_lycopersicum_chromosomes.4.00.fa; https://solgenomics.net/ftp/tomato_genome/assembly/build_4.00/S_lycopersicum_chromosomes.4.00.fa accessed on 7 August 2025). First, the genome index was constructed using hisat2-build with 8 threads (-p 8) in HISAT2 [22] (v2.2.1). Subsequently, reads from each sample were aligned using HISAT2 (parameters: -p 8 --dta -x) with 8 parallel threads to accelerate computation.

The resulting alignment files were sorted using samtools [23] (v1.22.1) and output as sorted BAM files for each sample. To evaluate alignment quality and yield, alignment statistics for each sample were generated using samtools flagstat, with metrics including the overall alignment rate, properly paired reads, and unmapped/duplicate reads. Post-alignment quality control was performed using the RSeQC package [24] (v5.0.4) to assess library specificity and RNA integrity. Specifically, the read_distribution.py, geneBody_coverage.py, and tin.py modules were employed to evaluate exonic mapping rates, 5′-to-3′ coverage uniformity, and Transcript Integrity Numbers (TINs), respectively. The sorted BAM files were subsequently used for expression quantification and differential expression analysis.

### 2.5. Differential Expression Analysis

Raw counts at the gene level were generated using featureCounts [25] (Subread v2.1.1). The gene annotation file (ITAG4.0_gene_models.gff) was first converted and validated using gffread [26] (v0.12.7). Subsequently, featureCounts was executed with parameters -T 8 -p --countReadPairs -t exon -g gene_id to assign aligned reads to genomic features, processing all sorted BAM files and generating the primary output file featurecounts.txt.

Post-processing of featurecounts.txt was performed to extract gene lengths and raw count values per sample. This included replacing table headers with concise sample identifiers and removing the “gene:” prefix from gene IDs to facilitate downstream analysis. The resulting gene length and raw count matrices were used for subsequent expression normalization and differential expression analysis.

Differential expression analysis was performed on the gene-level raw count matrix obtained from featureCounts using the DESeq2 [27] (v1.48.2) R package. The raw count matrix was converted into a DESeqDataSet object with the design formula ~condition (groups: CK, DC12, DC24). Low-expression genes were filtered out, retaining only those with counts ≥ 10 in at least two samples. The DESeq function was subsequently executed for normalization and differential analysis. Log2 fold changes (LFC) were shrunk using the ashr method.

For the pairwise comparisons DC12 vs. CK, DC24 vs. CK, and DC24 vs. DC12, differentially expressed genes (DEGs) were identified with the thresholds of adjusted *p*-value (padj) < 0.05 and absolute log2 fold change (|log2FoldChange|) ≥ 1 [28]. The following results were saved: normalized counts, differential analysis results, DEG lists (total, upregulated, downregulated), intersection and union lists of DEGs, and summary statistics. The background gene set consisted of all genes tested in the analysis.

Visualization was primarily conducted using the following methods: a sample distance heatmap and a PCA plot based on VST-transformed data were generated using the pheatmap (v1.0.13) [29] and ggplot2 [30] (v4.0.0) R packages, respectively; volcano plots highlighting up- and downregulated DEGs were created with the top 12 most significant genes annotated using the ggrepel package (v0.9.6); and heatmaps displaying DEGs based on VST Z-score normalization were produced using pheatmap (v1.0.13). All analyses were performed within the R environment, with package installation and management handled through BiocManager.

### 2.6. Enrichment Analysis

Gene Ontology (GO) and KEGG pathway enrichment analyses were performed using the clusterProfiler package [31] (v4.16.0). Solyc IDs were converted to Entrez IDs prior to analysis. GO enrichment was conducted on up- and downregulated DEGs separately for each comparison using the enrichGO function (ont = “ALL”, *p*-value cutoff = 0.05). KEGG pathway analysis was performed on all DEGs for each comparison using the enrichKEGG function (organism = “sly”). The top significant terms were selected based on adjusted *p*-values, and the results were visualized using ggplot2.

### 2.7. Plasmid Construction

To achieve overexpression of *SlNSP-like*, the coding sequence (CDS) of *SlNSP-like* (excluding the stop codon) was inserted into the pBin-GFP vector under the control of the CaMV 35S promoter to generate the *p35S::SlNSP-like:GFP* fusion vector.

### 2.8. Subcellular Localization

Subcellular localization assay was performed on leaves of 3-week-old *Nicotiana benthamiana*. The p35S::SlNSP-like:GFP expression plasmid and the p35S::GFP empty vector control were introduced into the Agrobacterium strain GV3101. Bacterial cultures carrying the expression plasmid and empty vector control were first cultured in LB liquid medium supplemented with kanamycin and rifampicin antibiotics for 16 h. Then, 1 mL of each culture was transferred into 50 mL of LB liquid medium containing the same antibiotics and cultured for an additional 16 h. Bacterial cells were harvested by centrifugation at 5000 rpm for 5 min. After discarding the supernatant, the pellet was resuspended in an appropriate volume of infiltration buffer (10 mM MES and 10 mM MgCl_2_, pH 5.6 adjusted, with acetosyringone (AS) added to a final concentration of 200 μM). The optical density at 600 nm (OD_600_) of the resuspended bacterial suspension was adjusted to 0.4. The prepared bacterial suspension was then used for leaf infiltration. Following infiltration, *N. benthamiana* plants were kept in darkness for 48 h. DAPI signals and GFP fluorescence were observed using a confocal laser scanning microscope (Zeiss, Jena, Germany) with a 20× objective lens, excited at 405 nm and 488 nm, respectively.

### 2.9. Gene Expression Analysis by qRT-PCR

Total RNA was isolated from frozen tomato leaf samples using RNAiso Plus (Takara, #9101) following standardized protocols. Quantitative real-time PCR (qRT-PCR) was employed to assess transcriptional changes in *Pst* infection-associated genes across different time points post-treatment. First-strand cDNA synthesis was performed using a dedicated cDNA Synthesis Kit (Vazyme, #R223-01) to generate templates for downstream analysis. qRT-PCR experiments were conducted on the Bio-Rad CFX96 system in technical triplicates using ChamQ SYBR qPCR Master Mix (Vazyme, #Q312-02) to ensure technical precision. Relative gene expression levels were calculated using the 2^−^^ΔΔCt^ method, with ACTIN serving as the internal reference gene for normalization, as previously described [32]. Data visualization was achieved through histograms generated using GraphPad Prism software, providing clear graphical representation of expression patterns. All experimental conditions included three biological replicates to validate data reliability, and primer sequences utilized in this study are detailed in Table S6.

### 2.10. Transient Overexpression Experiment of Tomato Leaves

Transient expression was carried out on the leaves of 3-week-old tomato seedlings. Briefly, 1 mL needleless syringe was used to slowly inject the bacterial solution into the lower epidermis on the back of the leaves. The injected plants were then placed in a light incubator for normal cultivation. Samples were collected 7 days after injection for qRT-PCR detection of *SlNSP-like* expression levels and other experiments.

### 2.11. Pst DC3000 Infection and Bacterial Counting

Pst DC3000 was cultured in PSA medium supplemented with 50 μg/mL rifampicin. For tomato infection assays, the bacterial culture was inoculated into 200 mL PSA medium and grown overnight at 28 °C with shaking. Bacterial cells were harvested by centrifugation at 3000 rpm for 10 min, washed, and resuspended in 10 mM MgCl_2_ containing 0.005% (*v*/*v*) Tween-20 to achieve an optical density (OD_600_) of 0.2. The prepared bacterial suspension was evenly sprayed onto tomato plants to ensure uniform inoculation.

To quantify bacterial colonization, leaf samples were processed as follows: Immediately after 2 h of inoculation, surface-attached bacteria were removed by rinsing leaves three times with sterile water, and these samples were used for initial (0 dpi) bacterial titer determination. The remaining plants were maintained in darkness for 24 h before returning to standard photoperiod conditions for continued growth. For bacterial enumeration, 6 mm diameter leaf discs were excised from the fifth true leaf of each plant using a sterile puncher. Each disc was homogenized in 10 mM MgCl_2_, and the resulting homogenates were serially diluted (10^−1^ to 10^−3^) in sterile MgCl_2_. In addition, 10 μL of each dilution was spread onto PSA plates containing 50 μg/mL rifampicin and incubated at 28 °C for 36 h. Bacterial colonies were counted to calculate colony-forming units (CFUs) per unit leaf area, providing a quantitative assessment of pathogen proliferation over time.

### 2.12. Statistical Analysis

Data points represent the mean ± standard deviation (SD) of three replicates. The statistical significance of differences in parameters measured between WT and transgenic plants was analyzed using Student’s *t*-test. Significant differences relative to the control are indicated by * *p* < 0.05, ** *p* < 0.01, and *** *p* < 0.001. The Tukey test was also used for statistical analysis. Columns labeled with different letters are significantly different (*p* < 0.05). The GraphPad Prism 10.2.1 software was used for statistical analysis.

## 3. Results

### 3.1. Phenotypic Changes in Tomato Leaves After Inoculation with Pst DC3000

**Figure 1:**
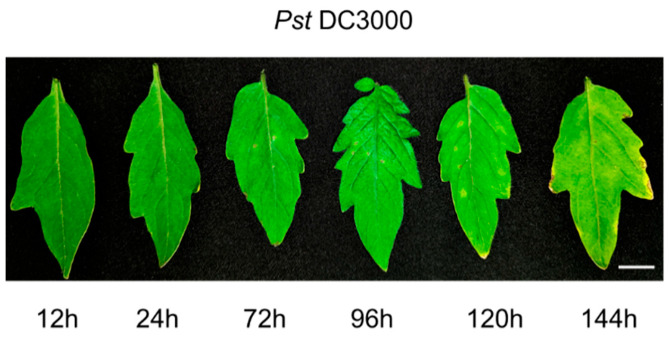
Tomato leaf symptoms at different time points after inoculation with *Pst* DC3000. The scale bar = 1 cm.

To investigate the impact of *Pst* DC3000 infection on tomato leaf phenotypes, uniformly sized and growth-stage-matched tomato plants were selected for inoculation under controlled greenhouse conditions. Leaf samples were systematically collected at multiple time points for phenotypic analysis. The results demonstrated that during the initial infection stages (12 h and 24 h), no significant morphological alterations were detected in the leaf tissues. Starting from 72 h after inoculation, however, characteristic yellow necrotic lesions began to emerge progressively across the leaf surfaces. With prolonged infection duration, these lesions expanded in both size and number, accompanied by a gradual loss of chlorophyll content, resulting in noticeable chlorosis (as shown in Figure 1).

### 3.2. Overview of the Transcriptome Libraries

In order to screen for early genes in tomato response to *Pst* DC3000 infection, we collected tomato leaf samples and performed transcriptome sequencing at relatively early time points 12 h and 24 h after inoculation. Each library was subjected to Illumina paired-end sequencing, generating a total of nine samples: CK_1, CK_2, CK_3, DC_12_1, DC_12_2, DC_12_3, DC_24_1, DC_24_2 and DC_24_3. A summary of the raw sequencing reads and quality filtering results is presented in Table S1. Quality trimming (PHRED score ≥ 20) and filtering (minimum read length = 25 nt) were performed to improve read quality, retaining approximately 99% of the raw reads on average. The total number of sequences per library ranged from 23 to 28 million, with an average of approximately 25 million, ensuring sufficient depth for RNA-seq analysis. Quality assessment revealed that the read length of all libraries was predominantly 150 bp. Under paired-end sequencing, the average total sequencing output per library was about 7.53 Gb, with abundant total bases and stable GC content within the range of 42–43%, as shown in Table S2. The mean PHRED quality score calculated by base position remained consistently high, with an overall average of 39.49. The average quality scores across all samples were concentrated in the high-quality region, exhibiting a unimodal and right-skewed distribution curve without a distinct low-quality peak, indicating stable overall sequencing data quality with minimal low-quality sequences. The high retention rate and consistent quality metrics collectively demonstrate that the sequencing data are of high quality and suitable for subsequent transcriptome quantification analysis.

The high-quality reads were aligned to the tomato reference genome (S_lycopersicum_chromosomes.4.00.fa). On average, 95.35% of the high-quality reads were successfully mapped to the reference genome. Among the mapped paired-end reads, an average of 87.30% were uniquely mapped, while 4.08% were aligned to multiple locations, and 8.63% of paired reads remained unpaired (Table S3). Additionally, post-alignment quality assessment using RSeQC confirmed the high integrity and specificity of the libraries, showing a mean TIN > 82 and an exonic mapping rate >93% across all samples (Table S3).

Using the sorted alignment files from each sample, we performed gene expression quantification to obtain read counts for each gene. This process yielded a gene count matrix containing the read counts of each gene across all samples, establishing a foundation for subsequent differential expression analysis and functional enrichment studies.

### 3.3. Transcriptome Differential Expression Analysis

**Figure 2:**
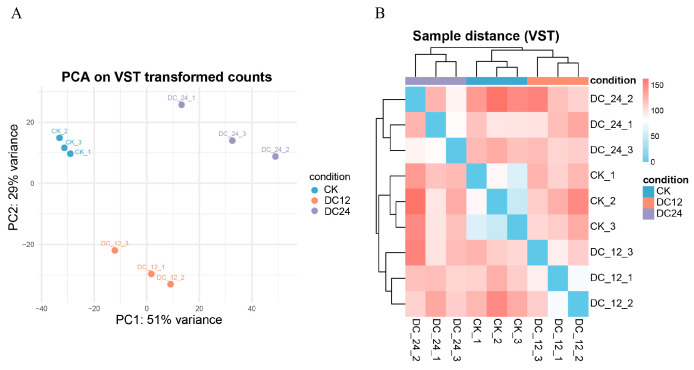
Principal component analysis and sample-to-sample distance of RNA-seq data. (**A**) Principal component analysis (PCA) plot showing the clustering of nine samples. The percentage of variance explained by PC1 and PC2 is indicated on the axes. Abbreviations: CK, control; DC12, 12 h post-inoculation; DC24, 24 h post-inoculation with *Pst* DC3000. (**B**) Sample-to-sample distance heatmap based on VST-transformed data. Colors indicate Euclidean distance, with blue representing higher similarity and red representing lower similarity.

For RNA-seq data analysis, differential expression analysis was performed on the gene count matrix using the DESeq2 package. The raw count data in the DESeqDataSet were transformed using a variance-stabilizing transformation (VST) for principal component analysis and sample distance analysis (Figure 2A). The results demonstrated clear separation among different condition groups (CK, DC12, and DC24) in the PCA space. Biological replicates within the same group (e.g., the three CK replicates) clustered closely together, while distinct differences were observed between the experimental groups (DC12 and DC24) and the control group (CK) (Figure 2B). The high consistency among biological replicates enhanced the reliability of the differential expression analysis conclusions.

**Figure 3:**
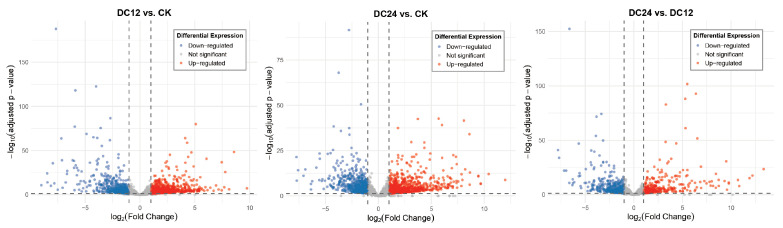
Volcano Plot of Differentially Expressed Genes between DC12 and CK, DC24 and CK, DC24 and DC12.

To visually represent the significance and direction of expression changes, volcano plots were generated for the DC12 vs. CK, DC24 vs. CK, and DC24 vs. DC12 (Figure 3) comparisons. These plots highlighted significantly upregulated (red) and downregulated (blue) genes. Based on the sample groupings (with the CK group as control and DC12/DC24 as treatment groups), low-expression genes were filtered out, retaining only genes with counts ≥10 in at least two samples. For the pairwise comparisons of DC12 vs. CK and DC24 vs. CK, differentially expressed genes (DEGs) were identified using the thresholds of adjusted *p*-value (padj) < 0.05 and absolute log_2_ fold change (|log_2_FC|) ≥ 1. The analysis revealed 2103 DEGs in the DC12 vs. CK comparison, consisting of 1267 upregulated and 836 downregulated genes. The DC24 vs. CK comparison identified 2055 DEGs, with 1211 upregulated and 844 downregulated genes. The DC24 vs. DC12 comparison detected 1044 DEGs, of which 484 were upregulated and 560 were downregulated. Among these, 15 were consistently upregulated and 9 were downregulated genes across all post-inoculation time points, while the union comprised 3823 DEGs, out of a total background of 21,782 analyzed genes (Figure S1).

In summary, the DESeq2-based differential expression analysis, supported by multiple visualization methods, consistently indicated that both DC12 and DC24 treatments significantly reshaped the transcriptome. These highly reproducible sets of upregulated and downregulated genes provide a reliable candidate list for subsequent functional enrichment analysis, pathway investigation, and experimental validation, thereby facilitating the elucidation of the molecular mechanisms underlying the treatments and the screening of potential biomarkers.

### 3.4. Enrichment Analysis

We performed Entrez ID mapping for six differentially expressed directions (DC12 vs. CK up, DC12 vs. CK down, DC24 vs. CK up, DC24 vs. CK down, DC24 vs. DC12 up, and DC24 vs. DC12 down), with mapping rates of 41.0%, 38.5%, 37.6%, 38.4%, 39.9%, and 43.6%, respectively. The full set of genes used in the differential expression analysis served as the background gene set, with a mapping rate of 39.4% (8586 genes successfully mapped out of 21,782 genes). After successful mapping, 520, 322, 455, 324, 193, and 244 differentially expressed genes from the aforementioned six groups (Table S4), respectively, were used for subsequent Gene Ontology (GO, including BP, CC, and MF) and KEGG pathway enrichment analyses, with the same 8586 successfully mapped genes serving as the background.

**Figure 4:**
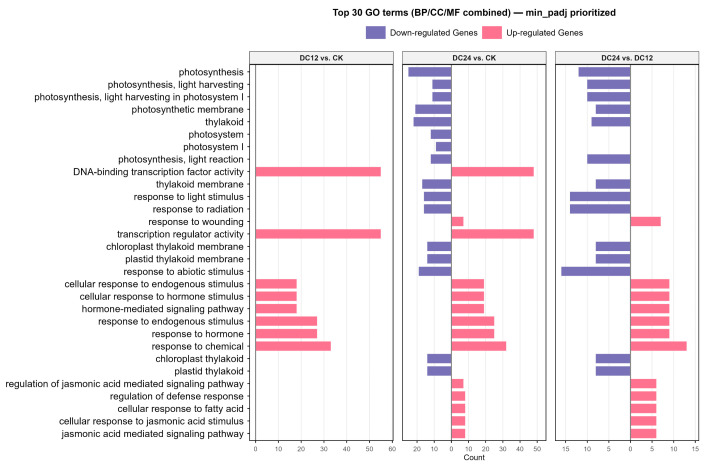
Top 30 GO terms from combined ontologies ranked by minimum adjusted *p*-value. The bar plot shows the count of differentially expressed genes for each term, with pink indicating upregulation and purple indicating downregulation.

Both GO enrichment analysis and KEGG pathway enrichment analysis were conducted separately for the up- and downregulated DEGs from each pairwise comparison. The numbers of significantly enriched GO terms (p adjusted < 0.05) were as follows: for the DC12 vs. CK upregulated group, there were 27, 4, and 23 significant terms in the Biological Process (BP), Cellular Component (CC), and Molecular Function (MF) ontologies, respectively; for the downregulated group, there were four and two significant terms in the BP and CC ontologies, respectively. For the DC24 vs. CK upregulated group, there were 61 and 17 significant terms in the BP and MF ontologies, respectively. For the downregulated group, there were 9, 23, and 10 significant terms in the BP, CC, and MF ontologies, respectively. For the DC24 vs. DC12 upregulated group, there were 47 and 7 significant terms in the BP and MF ontologies, respectively. For the downregulated group, there were 10, 19, and 5 significant terms in the BP, CC, and MF ontologies, respectively (Figure 4).

To comprehensively display the most representative and significant GO terms, we aggregated the GO enrichment results from the six comparison sets (DC12 vs. CK up/down, DC24 vs. CK up/down, DC24 vs. DC12 up/down). The GO terms were ranked primarily by their minimum adjusted *p*-value across the six lists (in ascending order). Secondary sorting was applied based on the frequency of occurrence of the term across the different lists (in descending order) in cases of ties. Finally, the top 30 GO terms (spanning the three ontologies: BP, CC, and MF) were selected and visualized using a faceted bar plot. Positive and negative bars represent upregulated and downregulated terms, respectively (Figure 4). Notably, we observed significant enrichment of terms such as DNA-binding transcription factor activity, hormone-mediated signaling pathway, and regulation of defense response, which are commonly associated with pathogen response mechanisms. Additionally, pathways including photosynthesis, light harvesting, and the jasmonic acid-mediated signaling pathway were recurrently enriched across multiple comparative analyses (Figure S2), indicating that pathogen infection induces broader physiological impacts beyond immediate disease resistance responses. These findings collectively highlight the interconnected nature of transcriptional regulation, hormonal signaling, and metabolic processes in orchestrating plant defense strategies while underscoring systemic physiological alterations triggered by pathogen invasion.

**Figure 5:**
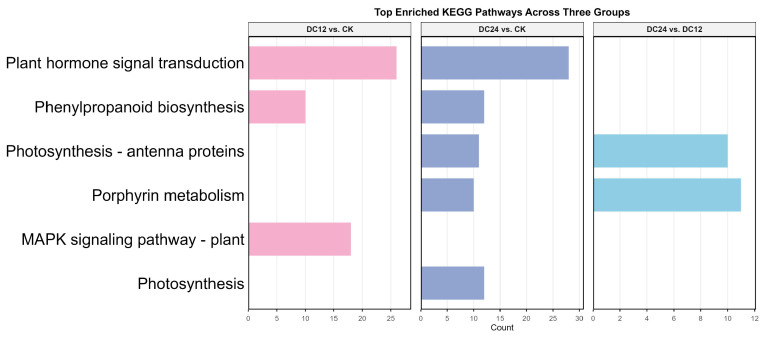
Enriched KEGG pathways across comparison groups. The bar charts display the number of differentially expressed genes annotated to each pathway. Colors represent the different comparison groups: pink (DC12 vs. CK), purple (DC24 vs. CK), and blue (DC24 vs. DC12). Only pathways with a Benjamini–Hochberg adjusted *p*-value < 0.05 are shown.

The KEGG enrichment bar chart (Figure 5) illustrates key pathways and their corresponding enrichment statistics. Although the number of significantly enriched pathways (*p* < 0.05) is relatively limited, the analysis identifies several metabolic and signaling pathways potentially modulated by the experimental treatments. Specifically, pathway enrichment analysis reveals: three for the DC12 vs. CK group, five for the DC24 vs. CK group, and two for the DC24 vs. DC12 group. Notably, these enriched pathways—without directional distinction of up/downregulation—include critical disease resistance-associated entries such as plant hormone signal transduction and the MAPK signaling pathway. The findings indicate that while the enrichment profile exhibits quantitative constraints, the identified pathways reflect systemic physiological adjustments and defense-related signaling cascades activated during pathogen infection.

### 3.5. Explore and Validate the Early Response Genes in Pst DC3000 Infection

**Figure 6:**
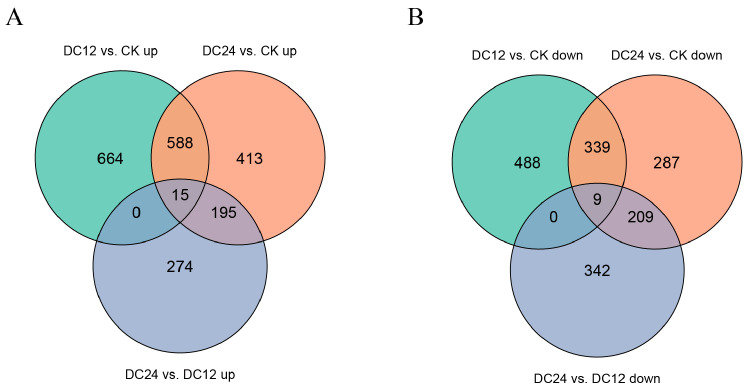
Venn diagram of DEGs across comparisons. The number of differentially expressed genes (upregulated (**A**) and downregulated (**B**)) compared between groups is listed in the figure.

The Venn diagram (depicted in Figure 6) visualizes the intersection of upregulated and downregulated differentially expressed genes (DEGs) across three pairwise comparisons. For upregulated DEGs, Venn diagram analysis revealed distinct overlap patterns: 588 genes were commonly upregulated in both DC12 vs. CK and DC24 vs. CK, while 413 genes were shared between DC24 vs. CK and DC24 vs. DC12, and 195 genes co-upregulated in DC12 vs. CK and DC24 vs. DC12. Notably, 15 genes demonstrated consistent upregulation across all three comparison groups. The number of uniquely upregulated genes specific to each comparison was 664 for DC12 vs. CK, 413 for DC24 vs. CK, and 274 for DC24 vs. DC12.

For downregulated DEGs, similar analysis showed 339 genes commonly downregulated in both DC12 vs. CK and DC24 vs. CK, 287 genes shared between DC24 vs. CK and DC24 vs. DC12, and 209 genes co-downregulated in DC12 vs. CK and DC24 vs. DC12. Only nine genes maintained consistent downregulation across all three comparisons. The uniquely downregulated genes were 488 for DC12 vs. CK, 287 for DC24 vs. CK, and 342 for DC24 vs. DC12, respectively.

### 3.6. Expressional Validation of the DEGs

**Figure 7:**
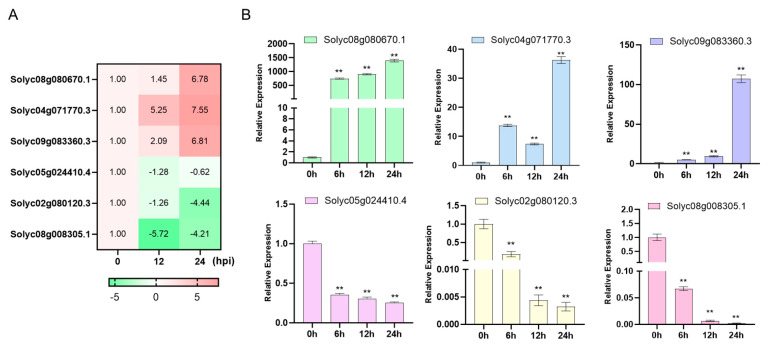
qRT-PCR verification of six candidate genes. (**A**) The six candidate genes heatmap of the transcriptome data. (**B**) Quantitative analysis of six candidate genes expression. *ACTIN* was used as the internal control. Data are presented as the mean ± SD of three biological replicates (** *p* < 0.01, Student’s *t*-test).

To assess the reliability of RNA-seq data, we conducted qRT-PCR analysis on a subset of six differentially expressed genes (DEGs). These DEGs were chosen from a pool of 15 genes that were consistently upregulated and 9 genes that were consistently downregulated throughout the infection process. Notably, these selected DEGs primarily play roles in “Plant hormone signal transduction” and “Plant–pathogen interaction,” with a significant portion being transcription factors (Figure 5). By comparing the expression patterns obtained from qRT-PCR with those from RNA-seq across various inoculated samples, we found a strong consistency between the two methods (Figure 7). This alignment confirms the accuracy of the RNA-seq analysis.

### 3.7. SlNSP-like Is an Important Gene Involved in Tomato Response to Pst DC3000

Among the candidate genes validated by fluorescence quantification, we noticed that the Solyc05g024410.4 gene showed significant downregulation 6 h after inoculation, indicating that this gene is an early gene in plant response to pathogen invasion. The sequence annotation shows that Solyc05g024410.4 encodes a nitrile specific protein-like (NSP-like) protein. Our previous research has shown that the NSP2 gene in *Arabidopsis* is also involved in resistance to *Pst* DC3000 [33], and there have been no functional reports of such genes in tomatoes. Therefore, we further investigated the function of the *SlNSP-like* gene.

**Figure 8:**
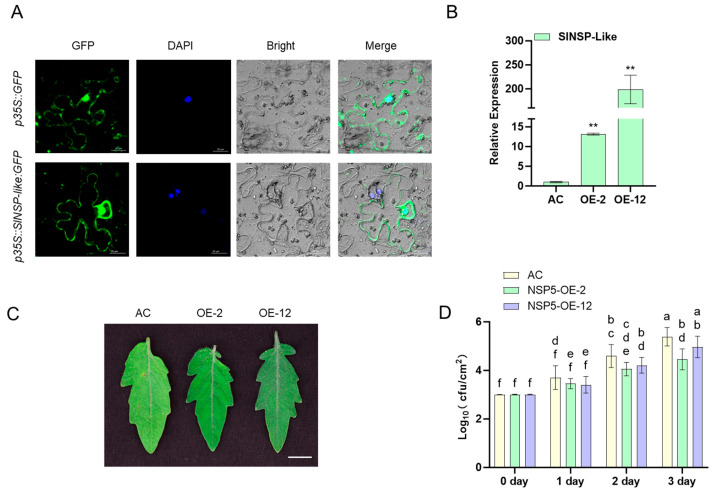
Functional analysis of *SlNSP-like* gene by transient expression in tomato plants. (**A**) Transient expression analysis in *Nicotiana benthamiana* leaves. The scale bar = 20 μm. (**B**) Quantitative analysis of *SlNSP-like* gene expression in transgenic tomato leaves. *ACTIN* was used as the internal control. Data are presented as the mean ± SD of three biological replicates (** *p* < 0.01, Student’s *t*-test). The assays were carried out at least three times, with analogous results obtained. (**C**) Phenotypic comparison of disease resistance in transgenic tomato lines 3 days post-inoculation with *Pst* DC3000. Bar = 1 cm. (**D**) Bacterial growth curve analysis at muti-time points. Data are presented as means ± SE (n = 3), *p* < 0.05 with two-sample *t*-test. Different letters represent different significance.

Transient expression of tobacco leaves shows that SlNSP-like protein is localized in the cytoplasm and nucleus (Figure 8A). Furthermore, we transiently expressed the constructed overexpression vector in tomato leaves, and the *SlNSP-like* expression levels of the two transformation lines increased by about 15-fold and 200-fold (Figure 8B), respectively. After inoculation, it was found that two overexpression lines exhibited significant disease resistance, and the leaves remained green after 3 days of inoculation, while the control leaves gradually faded (Figure 8C). The results of the bacterial growth curve also reflect a similar situation, with significantly lower leaf bacterial counts in the two overexpression lines on different time points after inoculation compared to the control (Figure 8D).

## 4. Discussion

This study utilized RNA-Seq technology to systematically profile transcriptomic dynamics in tomato plants at 12 h (DC12) and 24 h (DC24) post-DC3000 infection. Through an integrated bioinformatics pipeline, we identified substantial transcriptional reprogramming during pathogenesis (Figure 4). Key DEGs and associated pathways were characterized, providing critical molecular insights into tomato immune responses. Rigorous quality control confirmed high data reliability, with 95.35% average alignment to the tomato reference genome and consistent biological replicates, validated by PCA and distance heatmaps showing clear transcriptional segregation between control and infected groups (Figure 2).

Consistent upregulated and downregulated expression patterns across treatments were visualized through volcano plots and hierarchical clustering, demonstrating robust transcriptome remodeling during early infection (Figure 3). Among the 15 consistently upregulated and 9 downregulated genes identified across all post-inoculation time points (Table S5), we observed that the sustained upregulation of AP2/ERF domain-containing protein (Solyc04g071770.3) and bHLH domain-containing protein (Solyc09g083360.3) following pathogen inoculation indicates that plants employ transcription factors to initiate transcriptional reprogramming during the early stages of infection. Simultaneously, pathogenesis-related proteins such as PR1a (Solyc01g106620.2) and osmotin-like proteins (Solyc08g080660.1, Solyc08g080670.1) exhibited significant upregulation, underscoring their critical role as integral components of the plant’s early defense system [13]. This coordinated transcriptional response highlights the molecular strategies plants deploy to counteract pathogen invasion through rapid activation of defense-associated genes and regulatory networks.

GO enrichment analysis revealed significant enrichment of immune-related biological processes including “defense response” and “hormone-mediated signaling pathway,” alongside cellular components such as “plasma membrane” and “extracellular region,” and molecular functions like “kinase activity” and “DNA-binding transcription factor activity” (Figure 4). These results confirm the coordinated activation of defense pathways and transcriptional networks in tomato immune responses against DC3000 [34]. Notably, several previously characterized *Pst* resistance genes in tomatoes (e.g., *SlATL2*, *SlWRKY71*, *SlWRKY75*, *SlSAD2*, *SlβCA3*) [6,16,35,36,37], while operating through distinct signaling pathways, predominantly converge on established major pathways, suggesting that tomato resistance to *Pst* relies primarily on several conserved pathway frameworks.

KEGG pathway analysis identified crucial metabolic/signaling pathways, including “Plant-pathogen interaction”, “Phenylpropanoid biosynthesis”, and “Plant hormone signal transduction”. These pathways regulate defense metabolite biosynthesis and signaling networks (Figure 5). Especially, these pathways have been proven to be highly correlated with the plant immune system [38,39,40]. Notably, incomplete tomato genome annotation limited gene mapping to Entrez IDs [41], affecting enrichment comprehensiveness. Future studies should integrate multi-omics annotations and homology-based prediction to characterize unannotated genes.

Functional characterization of SlNSP-like protein revealed its dual localization in cytoplasm and the nucleus (Figure 8A), implying roles in transcriptional regulation and signaling. Overexpression in tomato leaves (15–200-fold increased expression) enhanced resistance to *Pst*, evidenced by reduced bacterial proliferation and sustained leaf vigor, contrasting with *Arabidopsis* NSP2 overexpression lines showing impaired resistance at 2 dpi [33]. This species-specific divergence establishes *SlNSP-like* as a positive regulator of tomato immunity, offering cross-species insights for breeding pathogen-tolerant Solanaceae crops.

NSP proteins were traditionally considered exclusive to cruciferous plants, with no functional reports in other taxa. Comparative genomics revealed 72 NSP family genes in *Brassica napus* (a cruciferous species) versus only 5 in *Arabidopsis* [42], suggesting underexplored functional complexity within this protein family. The discovery of SlNSP-like function in tomato disease resistance expands the understanding of NSP proteins beyond crucifers. This finding implies broader functional roles for NSP proteins in non-cruciferous plants and underscores the potential for conserved immune mechanisms across plant lineages. It opens avenues for novel genetic strategies to enhance pathogen resistance in diverse crop species, particularly in non-cruciferous families like Solanaceae, where such mechanisms may have been previously overlooked.

In summary, this integrated transcriptomic and bioinformatics study systematically elucidated dynamic gene-pathway alterations during tomato–DC3000 interactions. Key candidate genes and pathways provide a theoretical foundation for functional validation and resistance breeding. Specifically, a pathogen infection early response factor *SlNSP-like* has been isolated and preliminarily functionally identified, which will contribute to further detailed research on its mediating mechanism. Future research will prioritize functional characterization of core early response DEGs, particularly those consistently responsive across time points, to delineate precise molecular mechanisms within tomato immune signaling networks, advancing pathogen tolerance in Solanaceae crops through targeted genetic manipulation.

## 5. Conclusions

This study investigates tomato–*Pst* interactions through early-stage transcriptomic profiling to address knowledge gaps in molecular mechanisms. RNA-sequencing of nine libraries from *Pst* DC3000-infected tomato leaves revealed stage-specific transcriptional responses, with 484–1267 upregulated and 560–844 downregulated DEGs across time points. KEGG enrichment underscored metabolic reprogramming, plant–pathogen interaction networks, and hormone signaling cascades. Longitudinal analysis identified 15 consistently upregulated and 9 downregulated genes throughout the infection process. Functional validation highlighted SlNSP-Like, a cytoplasmic-nuclear protein homologous to *Arabidopsis AtNSP2*, as a novel resistance determinant; transient overexpression in tomato enhanced *Pst* DC3000 resistance. These findings elucidate critical regulatory nodes in tomato immunity, offering actionable targets for breeding disease-resistant cultivars and optimizing bacterial spot disease management strategies.

## Data Availability

The original contributions presented in this study are included in this article/Supplementary Materials. Further inquiries can be directed to the corresponding authors.

## Supplementary Materials

The following supporting information can be downloaded at: https://www.mdpi.com/article/10.3390/cimb48010011/s1.
