# Supplementary material for: Transcriptomics Analysis Reveals an Early Response Gene SlNSP-like Involved in Solanum lycopersicum Response to DC3000 Infection
Source: Curr Issues Mol Biol. 2025 Dec 22;48(1):11. doi: 10.3390/cimb48010011 (PMC12839917; doi:10.3390/cimb48010011)
Supplement: Supplementary file 1 [file cimb-48-00011-s001.zip › SUPP FIG.pdf]

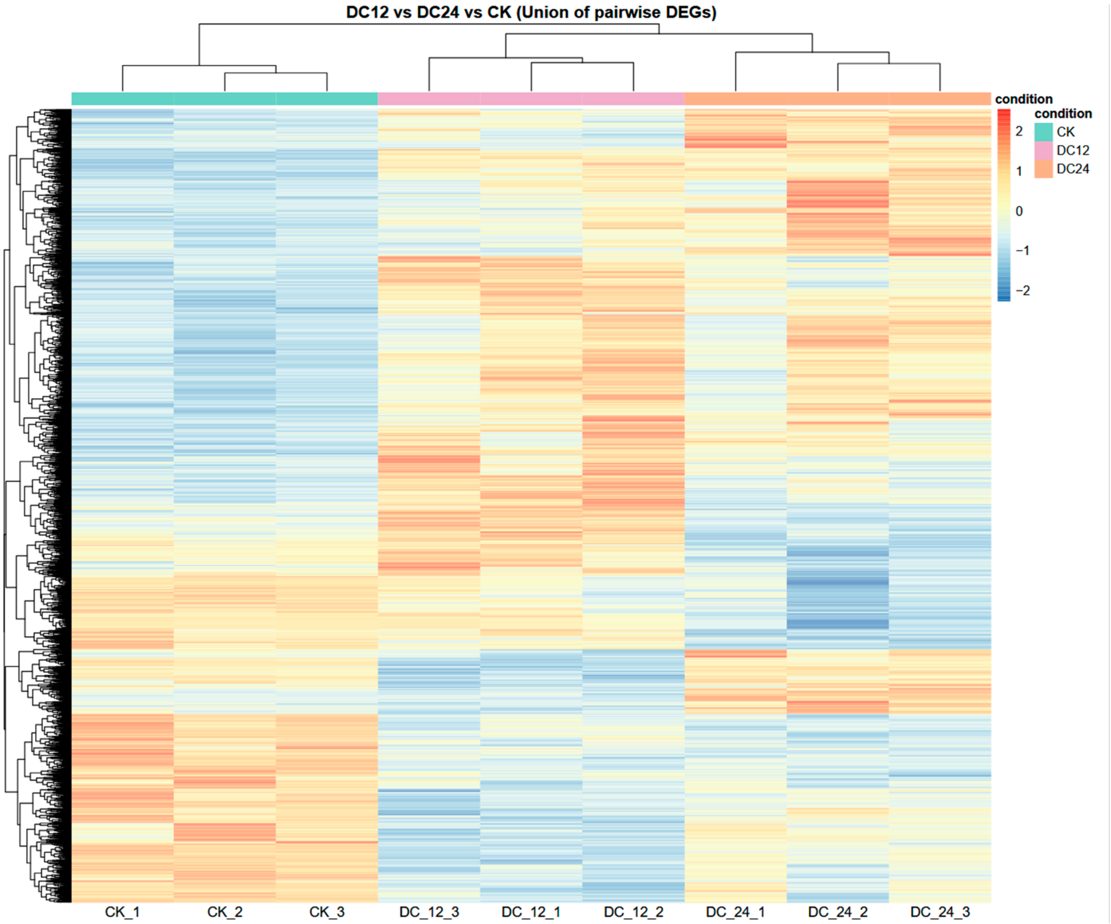

Figure S1. Union of DEGs from three pairwise comparisons (DC12 vs. CK, DC24 vs. CK, DC24 vs. DC12) across all samples.

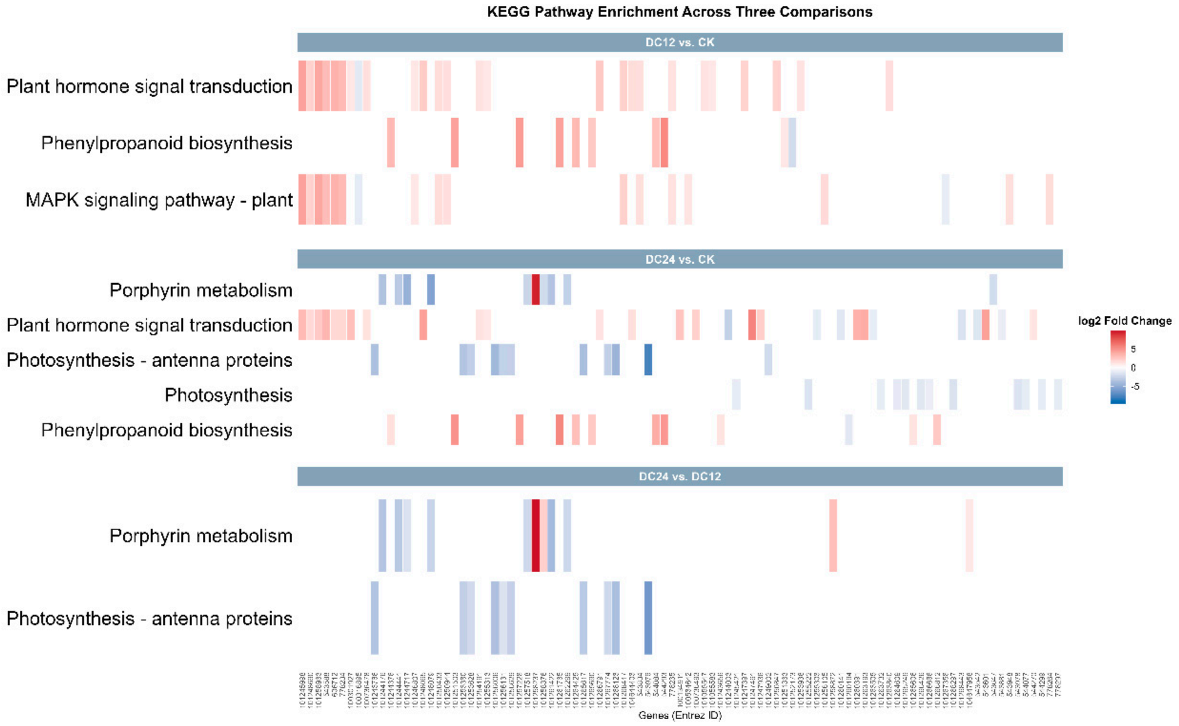

Figure S2. Heatmap of log2 fold change patterns for KEGG-enriched genes across three comparisons.
